# Effects of home-based interventions using exergames on physical and cognitive functions in community-dwelling older adults: a PRISMA-P-compliant protocol for a systematic review

**DOI:** 10.3389/fpubh.2023.1291120

**Published:** 2024-01-11

**Authors:** Julia Seinsche, Eling D. de Bruin, Timo Hinrichs, Eleftheria Giannouli

**Affiliations:** ^1^Movement Control and Learning Group, Institute of Human Movement Sciences and Sport, Department of Health Sciences and Technology, ETH Zürich, Zürich, Switzerland; ^2^Department of Health, OST—Eastern Swiss University of Applied Sciences, St. Gallen, Switzerland; ^3^Department of Neurobiology, Care Sciences and Society, Karolinska Institutet, Huddinge, Sweden; ^4^Division of Sports and Exercise Medicine, Department of Sport, Exercise and Health, University of Basel, Basel, Switzerland

**Keywords:** older adults, telerehabilitation, home setting, serious games, motor-cognitive training, physical activity, cognition, systematic review

## Abstract

**Introduction:**

Physical activity and exercise are crucial to counteract physical and cognitive decline in old age. Home-based exergame training can be a solution to overcome physical inactivity. This systematic review aims to provide a comprehensive overview of home-based exergame interventions and evaluate their effectiveness in improving cognitive and physical functions through physical activity enhancement in older adults.

**Methods and analysis:**

We are conducting a systematic literature search including studies examining (1) community-dwelling older adults aged 60 years and older without any specific disease, (2) exergame-based exercise programs that take place at least partially in a home setting, and (3) intervention-related physical and/or cognitive outcomes. We will include randomized controlled trials and any other type of pre-post study published in English. There are no restrictions in terms of control group type and publication date. A search string was created and used in PubMed, Web of Science, Embase, Scopus and CINAHL. In addition, a hand search is carried out. This involves checking the references of the included studies and searching Google Scholar for further studies. The included studies will be summarized and, if homogeneity is sufficient, a random-effects meta-analysis will be performed. We will assess the risk of bias using RoB 2.0 and ROBINS-I.

**Conclusion:**

The findings of this systematic review will help to define the most suitable exergame programs to counteract cognitive and physical decline in older adults. Additionally, they will inform the development of effective home-based exergame systems and point to future pathways of digital rehabilitation in older adults.

**Registration:**

Prospero (ID: CRD42023374234).

## 1 Introduction

The aging process is associated with declines in both physical and cognitive systems (1) impacting the ability to master activities of daily living and maintain independence (2). Physical activity [including structured exercise (3, 4)] has well known positive effects not only on physical functions (e.g., cardiovascular benefits, body composition, functional capacity, balance, and stability) but also transfer effects on cognition (e.g., on verbal and spatial memory, executive functions, the rate of cognitive decline, and the onset of neurodegenerative disorders such as dementia) (1, 5–10). For that reason, the WHO is recommending regular physical activity for older adults (11).

Despite these recommendations, the global age-standardized prevalence of physical inactivity is 27.5% with older adults being more likely to be physically inactive (12). The most frequently reported barriers to physical activity for older adults are: (1) health issues, (2) lack of company, (3) lack of interest, (4) limited accessibility, (5) a person’s fear (e.g., fear of going outside, injury, or falling), (6) individual preferences, and (7), lack of social support (13, 14).

Moreover, even among those who overcome these barriers, adherence to exercise programs is often low. In their review, Nyman et al. (15) found adherence values between 52% (for individually tailored exercise) to ≥70% (for walking and class-based exercise) in fall prevention interventions for older adults. Furthermore, they found that there was a tendency for adherence to decline over time.

Home-based exergame training can potentially address most of the barriers for physical activity in older adults. Pirovano et al. (16, p. 56) described exergames as “an exercise with a game built into its structure” and therapeutic exergames as “an exergame that supports all primary (i.e. elicit a given movement) and secondary goals (i.e. movement correctness) defined for an exercise.” Exergame training can easily be integrated into home settings, where it has already proven to be an effective tool for exercise interventions and rehabilitation in different populations of older adults (17, 18). Generally, compared to conventional exercise interventions, exergame training presents several advantages. Due to their game features, exergames can enhance enjoyment and engagement (19) which in turn might have a positive effect on adherence rates (20) and, thus, also on health and wellbeing (19). Furthermore, one unique feature of exergames is that they enable simultaneous physical and cognitive training, thus targeting physical and cognitive functions at the same time, which has shown to be equal or even superior to a separate or sequential training of both functions (21–24). Previous research has demonstrated the positive impact of exergames on cognitive functions (25, 26), physical functions (27) and psychological outcomes (25).

Generally, home-based training offers numerous benefits compared to conventional face-to-face rehabilitation including higher adherence (28), improved self-management, higher accessibility, lower costs, convenience, and easier integration into everyday life (29–32) while at the same time it is equally effective (33, 34). Furthermore, home training following inpatient rehabilitation contributes to covering the whole continuum of care (till a full recovery) because inpatient rehabilitation programs can seamlessly transition to a home setting—with or without prescription for outpatient rehabilitation, but in most cases, supervised remotely by therapists. Thereby, exergames are especially suitable to be conducted without direct supervision since most exergame systems provide immediate feedback. Meulenberg et al. concluded that exergames in the home-environment are effective and “promising options to overcome barriers of accessibility, discontinuity, and lack of resources” (35, p. 2). Given the growing number of home-based exergame programs and the corresponding increase in research done on them, a comprehensive overview is necessary to summarize the current state of evidence. Previous similar reviews investigated home-based exergame interventions for specific patient populations such as people with Parkinson (18), dementia and mild cognitive impairment (36), chronic stroke (37), multiple sclerosis (38), post-stroke disorder, COPD, post-knee surgery (39), or mixed populations of older adults (40–42) and/or focused only on effects on either physical (38, 41, 43, 44) or cognitive outcomes (17). However, home-based exergames ideally ought to be effective not only for older adults with specific health conditions but for all older adults, aiming to prevent the age-related declines in both cognitive and physical functions described above.

Therefore, the objective of this review is to systematically search available literature investigating various home-based exergame interventions targeting physical as well as cognitive outcomes in community-dwelling older adults without any specific health conditions, and to evaluate their effectiveness in improving those functions compared to all kinds of controls. More specifically, this review seeks to address the following research questions:

1. What types of home-based exergame interventions are used to improve cognitive and physical functions in community-dwelling older adults without specific health conditions?
2. How effective are those interventions in improving cognitive and physical functions in community-dwelling older adults?
3. What clinical (e.g., study population characteristics, interventions, and outcomes) and methodological (e.g., study design and risk of bias) characteristics explain the heterogeneity in the results?

## 2 Materials and methods

We will use the Preferred Reporting Items for Systematic Review and Meta-Analysis Protocols (PRISMA-P) (45) as a guide for the completion and reporting of the systematic review protocol which was preregistered in the International Prospective Register of Systematic Reviews (PROSPERO; ID: CRD4202337423).

### 2.1 Eligibility criteria

Inclusion and exclusion criteria were defined following the PICO framework (Participants, Intervention, Comparisons, Outcomes, and study design).

#### 2.1.1 Population

We will include studies investigating community-dwelling older adults aged 60 and older. Studies involving people residing in nursing homes will be excluded. Participants of included studies may suffer from common age-related diseases such as cognitive decline, arthrosis, osteoporosis, hypertension. However, studies targeting specific diseases with interventions designed exclusively for rehabilitating the respective patient population will be excluded. We will include studies with participants of all physical activity levels. Thus, studies with physically active as well as sedentary older populations will be eligible for inclusion.

#### 2.1.2 Intervention

We will consider all studies investigating exergame-based training programs which at least partially take place in a home-setting and aim to improve physical and/or cognitive functions. Studies which used exergames solely as a supplement to conventional therapy will be excluded. Studies investigating digital interventions without any game features (e.g., online fitness classes) will not be considered either.

#### 2.1.3 Comparison

Studies with an active or passive control group, as well as those without any control group, will be eligible for inclusion.

#### 2.1.4 Outcomes

To be included, studies should have objectively measured intervention-related changes in at least one of the following cognitive or physical performance parameters using a clearly reported and validated test. Outcomes based solely on therapist observations will be excluded.

##### 2.1.4.1 Physical outcomes

1. Lower extremity strength and/or power
2. Upper extremity strength and/or power
3. Balance
4. Endurance/aerobic capacity
5. Functional mobility
6. Gait

##### 2.1.4.2 Cognitive outcomes

1. Global cognitive performance
2. Executive functions like inhibition, cognitive flexibility, and working memory
3. Attention/attentional functions
4. Visuospatial skills
5. Learning and memory

#### 2.1.5 Study design

In this systematic review, we will include any intervention studies such as randomized controlled trials (RCTs), quasi-randomized trials, pilot studies, feasibility studies and any type of study with physical or cognitive outcomes as a pre-post assessment. However, we will exclude theses and non-original studies (e.g., poster, study protocols, or reviews).

The articles should be in English, but there are no restrictions regarding publication dates.

### 2.2 Information sources

A systematic literature search was already conducted in PubMed, Web of Science, Embase, Scopus, and CINAHL. To ensure literature saturation, we will also perform a hand search, i.e., search for additional studies in google scholar and scan reference lists of included studies or similar reviews search. Finally, other studies known to the authors will also be reviewed.

### 2.3 Search strategy

A variety of terms describing the intervention (content and setting), the outcomes, and the population have been collected resulting in a PubMed search strategy displayed in Table 1.

**Table 1:** Search strategy as applied in PubMed.

Outcome of interest	(motor* [tiab] OR cognition [Mesh] OR cogniti* [tiab] OR motor skills [Mesh] OR motor-cognitive [tiab] OR “executive function*” [Mesh] OR “muscle strength/physiology” [Mesh] OR “muscle power” [tiab] OR balance [tiab] OR endurance [tiab] OR mobility [tiab] OR gait [Mesh] OR “functional fitness” [tiab] OR “physical capacit*” [tiab] OR “functional capacit*” [tiab] OR cognition [Mesh] OR cogniti* [tiab] OR SPPB [tiab] OR attention [tiab] OR memory [tiab] OR visuospatial* [tiab])
	AND
Intervention type	(exergaming [MeSH] OR game* [tiab] OR gami* [tiab] OR exergam*[tiab] OR digital [tiab] OR “virtual reality” [tiab] OR VR [tiab] OR digital [tiab] OR internet [tiab] OR computer [tiab] OR “video gam*” [tiab] OR videogam* [tiab] OR ICT [tiab] OR “active video games” [tiab] OR “active video game*” [tiab] OR gerontechnology [tiab] OR “serious gam*” [tiab] OR xbox [tiab] OR Kinect [tiab])
	AND
Intervention setting	(telerehabilitation [Mesh] OR “tele-rehabilitation” [tiab] OR telerehabilitation [tiab] OR tele-exercise [tiab] OR “home-based” [tiab] OR home [tiab] OR “home-setting” [tiab] OR “home-environment” [Mesh] OR “at home” [tiab] OR “in-home” [tiab] OR unsupervised [tiab] OR independent* [tiab])
	AND
Target population	(“older adult*” [tiab] OR “older person*” [tiab] OR elder* [tiab] OR senior* [tiab] OR Aged [Mesh] OR “Aged, 80 and over” [Mesh] OR aging [Mesh] OR” community-dwelling” [tiab] OR „independently living “[tiab])

### 2.4 Study records

#### 2.4.1 Data management and selection process

Retrieved searches will be directly imported in Rayyan (46).^1^ After uploading the retrieved search results, the software identifies duplicates which we then control and remove. Afterwards, two reviewers will independently screen the studies with regard to in- and exclusion criteria in two screening rounds. First, studies will be in- or excluded based on their titles and abstracts. Afterwards, in a second screening round, remaining studies will be checked in a full text review. During screening, the blinded review option provided by Rayyan will be turned on to ensure unbiased screening. This option will be only deactivated after each screening round for the two reviewers to compare their results. In case of disagreement on inclusion, a third reviewer will be consulted, and the reviewers will discuss their decisions until consensus is reached. Neither of the reviewers will be blinded to journal titles, study authors, or institutions.

Reasons for exclusion are cited for each study only in full text screening. Therefore, a list was created ordering these reasons: (1) no full text, (2) wrong language, (3) wrong intervention, (4) wrong setting, (5) wrong outcome, (6) wrong population, (7) wrong study design, and (8) wrong publication type. The first reason of this order which is found to be applicable is indicated as reason for exclusion.

#### 2.4.2 Data collection and items

Each reviewer will extract data from the full texts of included studies and enter them manually into *a priori* designed tables separating physical from cognitive outcomes (Supplementary Table 1). Afterwards, all entered data will be cross checked with discrepancies resolved by consensus. To reduce the influence of reviewer experience, the data extraction form has been explained to and discussed with all reviewers in a joint meeting. Furthermore, data extraction will be conducted very carefully to avoid the inclusion of multiple reports on the same study, or overlapping results, respectively.

The following data will be extracted:

1. Study characteristics: name of first author, year of publication, study design and setting.
2. Study population characteristics: number of participants, health status, age, gender, and living situation.
3. Interventions: type of exergame and training parameters based on FITT-VP principles (frequency, intensity, time, type, volume, and progression), amount and type of supervision (technical implementation), and if applicable, interventions of the control group.
4. Outcomes: characteristics of physical and cognitive assessments (i.e., physical or cognitive domain, assessment tool), primary outcome if other.
5. Main Findings with respect to training related changes in physical and cognitive performance.

In case of missing information, the authors of the respective studies will be contacted and asked to provide this information.

### 2.5 Risk of bias in individual studies

Following recommendations by Büttner et al. (47), we will assess the risk of bias of included studies using RoB 2.0 (48) for randomized and ROBINS-I (49, 50) for non-randomized studies. RoB 2.0 comprises 5 domains: (1) bias arising from the randomization process, (2) bias due to deviations from intended interventions (in this study the effect of assignment to the intervention will be evaluated), (3) bias due to missing outcome data, (4) bias in measurements of the outcome, and (5) bias in selection of the reported result. The 7 domains of ROBINS-I are (1) bias due to confounding, (2) bias in selection of participants into the study, (3) bias due to classification of interventions, (4) bias due to deviations from the intended interventions, (5) bias due to missing data, (6) bias in measurements of the outcomes, (7) bias in selection of the reported results. All included studies will be evaluated on domain level and summary scores will be derived representing the lowest level assessed in any of the domains (48, 49).

### 2.6 Data synthesis

A systematic narrative synthesis of all data described in section 2.4.2 will be performed to explore the findings across the different trials. The results will be split according to the main outcome of each trial. We will then categorize results by primary outcome of the systematic review which is the type of intervention. Thus, if multiple studies for specific interventions exist, data will then be grouped accordingly to form a clear descriptive summary. Within these groups, if possible, we will present results in order of intervention duration, and level of risk of bias. Thus, we will include studies of any level of risk of bias but will consider the risk of bias when summarizing the results.

Meta-analyses will only be conducted in case of low clinical and methodological heterogeneity defined as “variation in study population characteristics, coexisting conditions, (co)interventions, and outcomes evaluated across studies included,” and “variability in study designs and risk of bias,” respectively (51, p. 38), (52, 53) and only RCTs will be included. At this stage, the assessment of heterogeneity can only be done qualitatively.

If meta-analyses are performed, we will use ReviewManager (RevMan, Version 5.4, The Cochrane Collaboration, 2020). Expecting a certain amount of heterogeneity, we will conduct meta-analyses using a random-effect model (54). Concerning effect sizes, we will calculate Hedge’s g (standardized mean differences) for between-group comparisons. For this calculation, mean change scores from baseline to post measurement of each group, the respective standard deviations, and the number of participants will be used (53).

If the change-from-baseline standard deviations are not provided by the respective study authors, a correlation coefficient will be used for imputation, which, if possible, will be calculated from another study included in the meta-analysis or imputed from elsewhere.

Statistical heterogeneity of studies (variability in treatment effects) will be assessed using I^2^ statistic. Based on Higgins et al. (53) proposing a rough guide for interpretation, we will consider I^2^ values of 75% as considerable heterogeneity. If high levels of statistical heterogeneity are detected (I^2^ ≥ 75%), it can, but does not have to be caused by clinical and methodological heterogeneity (51) which is why we will perform a subgroup and/or a sensitivity analysis, respectively, aiming to find an explanation for this. Subgroup analyses will be based on (1) participant characteristics (age, sex), (2) types of intervention (to be determined, e.g., commercial games vs. games created for the specific population, or comparison of different exergame devices), (3) duration of the intervention period, and (4) exact physical or cognitive outcomes, respectively. Details concerning the sensitivity analysis cannot be specified at this stage because “there are many decision nodes within the systematic review process that can generate a need for a sensitivity analysis” (53, p. 278). For example, a sensitivity analysis might be performed excluding studies with a high risk of bias or those whose control characteristics differ significantly.

### 2.7 Meta-bias(es)

Possible publication bias will be analyzed through visual inspection of funnel plots (54). Additionally, if study protocols of included trials are available, we consider comparing reported outcomes to investigate possible outcome reporting bias.

## 3 Conclusion

This systematic review will provide a comprehensive overview of home-based exergame interventions for community-dwelling older adults helping to select the most suitable and effective exergame programs to counteract age-related cognitive and physical declines. In addition, the results will contribute to the development of highly effective home-based exergame systems and point to future pathways for digital rehabilitation in older adults. Therefore, we expect the results to be of interest for practice and policy makers as well as for future research.

## Author contributions

JS: Conceptualization, Methodology, Writing – original draft. EB: Writing – review & editing. TH: Writing – review & editing. EG: Conceptualization, Methodology, Supervision, Writing – review & editing.

## Footnotes

^1^ http://rayyan.qcri.org
