# Supplementary material for: Effects of home-based interventions using exergames on physical and cognitive functions in community-dwelling older adults: a PRISMA-P-compliant protocol for a systematic review
Source: Front Public Health. 2024 Jan 11;11:1291120. doi: 10.3389/fpubh.2023.1291120 (PMC10808653; doi:10.3389/fpubh.2023.1291120)
Supplement: Supplementary file 1 [file Table_1.DOCX]

# Supplementary Table 1. Data extraction table

| **Study characteristics** | | **Study population** | **Interventions** | | | | **Outcomes** | | **Results** |
| --- | --- | --- | --- | --- | --- | --- | --- | --- | --- |
| **Study**  (Author, year) | **Study design** | **Study participants**  (Number, health status, age, gender, living situation) | **Type of Exergame**  (FITT-VP principles) | **Amount and type of supervision** | **Adherence** | **Control group**  (Intervention, if applicable) | **Outcome and outcome measure** | **Primary outcome**  (If other) | **Main findings** (Changes in physical/  cognitive performance) |
|  |  |  |  |  |  |  |  |  |  |
|  |  |  |  |  |  |  |  |  |  |
|  |  |  |  |  |  |  |  |  |  |
